# Unveiling the Efficacy of Sesquiterpenes from Marine Sponge *Dactylospongia elegans* in Inhibiting Dihydrofolate Reductase Using Docking and Molecular Dynamic Studies

**DOI:** 10.3390/molecules28031292

**Published:** 2023-01-29

**Authors:** Abdelsattar M. Omar, Khadijah A. Mohammad, Ikhlas A. Sindi, Gamal A. Mohamed, Sabrin R. M. Ibrahim

**Affiliations:** 1 Department of Pharmaceutical Chemistry, Faculty of Pharmacy, King Abdulaziz University, Jeddah 21589, Saudi Arabia; 2 Department of Pharmaceutical Chemistry, Faculty of Pharmacy, Al-Azhar University, Cairo 11884, Egypt; 3 Center for Artificial Intelligence in Precision Medicines, King Abdulaziz University, Jeddah 21589, Saudi Arabia; 4 Department of Biology, Faculty of Science, King Abdulaziz University, Jeddah 21589, Saudi Arabia; 5 Department of Natural Products and Alternative Medicine, Faculty of Pharmacy, King Abdulaziz University, Jeddah 21589, Saudi Arabia; 6 Preparatory Year Program, Department of Chemistry, Batterjee Medical College, Jeddah 21442, Saudi Arabia; 7 Department of Pharmacognosy, Faculty of Pharmacy, Assiut University, Assiut 71526, Egypt

**Keywords:** dihydrofolate reductase, sesquiterpenes, *Dactylospongia elegans*, industrial development, molecular docking, molecular dynamics, health and wellbeing

## Abstract

Dihydrofolate reductase (DHFR) is a crucial enzyme that maintains the levels of 5,6,7,8-tetrahydrofolate (THF) required for the biological synthesis of the building blocks of DNA, RNA, and proteins. Over-activation of DHFR results in the progression of multiple pathological conditions such as cancer, bacterial infection, and inflammation. Therefore, DHFR inhibition plays a major role in treating these illnesses. Sesquiterpenes of various types are prime metabolites derived from the marine sponge *Dactylospongia elegans* and have demonstrated antitumor, anti-inflammation, and antibacterial capacities. Here, we investigated the in silico potential inhibitory effects of 87 *D. elegans* metabolites on DHFR and predicted their ADMET properties. Compounds were prepared computationally for molecular docking into the selected crystal structure of DHFR (PDB: 1KMV). The docking scores of metabolites **34**, **28**, and **44** were the highest among this series (gscore values of −12.431, −11.502, and −10.62 kcal/mol, respectively), even above the co-crystallized inhibitor SRI-9662 score (−10.432 kcal/mol). The binding affinity and protein stability of these top three scored compounds were further estimated using molecular dynamic simulation. Compounds **34**, **28**, and **44** revealed high binding affinity to the enzyme and could be possible leads for DHFR inhibitors; however, further in vitro and in vivo investigations are required to validate their potential.

## 1. Introduction

DHFR (Dihydrofolate reductase) is a substantial enzyme that is accountable for the conversion of DHF (7,8-dihydrofolate) to THF (5,6,7,8-tetrahydrofolate), as well as folate to DHF in the presence of NADPH (nicotinamide adenine dinucleotide phosphate) [1]. This enzyme exists in various organisms, such as humans, plants, animals, and bacteria [2]. It is needed for maintaining THF, which is essential for carbon atom donation in the synthesis of pyrimidines, purines, and amino acids (methionine, glycine, N-formyl-methionyl tRNA, and serine). Thus, it is crucial for proper cellular proliferation and growth. Its prohibition leads to pyrimidine and purine starvation, which consequently stops DNA and RNA synthesis, resulting in cell death [3]. DHFR is a remarkable target for folate antagonists that are known as valuable therapeutic agents for inflammatory, neoplastic, parasitic, and infectious illnesses [4,5,6,7]. For example, it is targeted by anticancer agents such as methotrexate, which is renowned for treating leukemia, rheumatoid arthritis, osteosarcoma, lymphoma, and breast and lung cancers [5,8], as well as by antimalarial (e.g., proguanil and pyrimethamine) [7] and antibacterial (e.g., trimethoprim) drugs [4,9].

Owing to the growing drug resistance to some of the available DHFR inhibitors, research for the discovery of new and selective DHFR inhibitors has been intensely increased, using molecular modeling, synthetic techniques, in vivo and in vitro biological investigation, mechanistic studies, and structure-activity relationships [10,11,12].

Computer-aided drug design (CADD), including molecular design, molecular modeling, computational chemistry, and rational drug design, is a contemporary computational tool that is utilized in drug discovery methods for identifying and developing potential leads in silico, leading to a decrease in the number of metabolites to be experimentally assessed [13]. These methods have made crucial contributions to the development of drugs that are in clinical trials or have clinical uses. In this tool, diverse programs and software are utilized to generate and filter a set of compounds based on specific criteria, predict their physicochemical characters, predict proper targets, and evaluate their binding affinity to the predicted targets. SBDD (structure-based drug design) is a category of CADD that uses the 3D structure of the target to perform molecular dynamic simulation (MD) and docking investigations. Docking assessed the strength of compound-target binding; however, MD estimated the ligand-protein complex behavior and stability in aqueous circumstances to simulate the physiological condition [14].

The marine environment is a promising source of valuable natural metabolites, many of which are launching in the market or being evaluated in clinical trials as new drugs, particularly for cancer treatment and antimicrobials [15,16,17]. Recently, molecular docking has become a substantial tool in marine drug research worldwide for screening various marine metabolites to predict their possible bioactivities and mechanisms of action because of its relatively uncomplicated procedures [18,19].

Among marine sponges, *Dactylospongia elegans* (*D. elegans*) has been demonstrated to be a rich source of diverse metabolites with substantial bioactivities, including sesquiterpenes such as hydroquinone, quinone, and tetronic acid derivatives. The sesquiterpene hydroquinone/quinone family of terpenoids possessing a drimane or a rearranged drimane framework is a distinguished type of metabolite that is a result of mixed biosynthesis. They feature a bicyclic sesquiterpene moiety linked to a quinol or quinone moiety [20]. In our continued research interest to discover untapped bioactivities and underpinning mechanisms of the marine reported metabolites using SBDD, 87 sesquiterpene derivatives reported from *D. elegans* were screened for their DHFR inhibitory potential using molecular docking. Additionally, the highly ranked metabolites were further assessed by MD simulation.

## 2. Results and Discussion

### 2.1. Molecular Docking Evaluation

**Figure 1:**
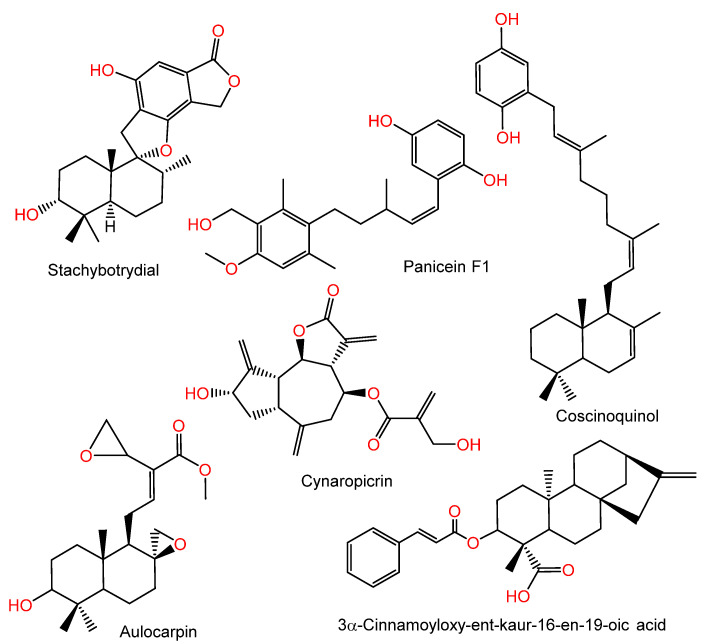
Structures of stachybotrydial, panicein F1, coscinoquinol, cynaropicrin, 3α-cinnamoyloxy-ent-kaur-16-en-19-oic acid, and aulocarpin.

Diverse natural metabolites of various structural classes, including terpenoids, have been assessed for their DHFR inhibition (DHFRI) capacity using in silico or in vitro approaches [21,22,23,24]. For example, cynaropicrin is a sesquiterpene lactone reported from artichoke that has notable DHFRI potential (IC_50_ 7.1 µM) [24]. Herrera-Acevedo et al. reported that the kaurane-type diterpene, 3α-cinnamoyloxy-ent-kaur-16-en-19-oic acid, possessed a high binding affinity to *Lb*DHFR-TS (*Leishmania panamensis* dihydrofolate-reductase-thymidylate-synthase), which is an important target for *L. panamensis* [25]. Further, in a docking study by Kumar et al., labdane diterpenoid-derived aulocarpin purified from *Afromomum aulocarpus* seed displayed *Plasmodium* wild-type DHFR antagonistic activity (Figure 1) [21].

Furthermore, some studies have reported the DHFRI potential of terpenoids containing the quinone moiety; for example, stachybotrydial is a drimane sesquiterpene containing a dioxgenated isobenzofuranone moiety, as reported by Kwon et al. from the soil derived *Stachybotrys* sp. FN298. It demonstrated *S. aureus`s* DHFR (IC_50_, 41 µM) inhibition potential and prohibited MRSA growth *(*MIC, 32 µg/mL), suggesting its possible efficacy as an antibacterial agent versus MRSA [26]. Coscinoquinol purified from the Australian *Coscinoderma* sp. sponge had cytotoxic potential versus P-388, A-549, HT-29, and CV-1 (IC_50_ values of 0.25, 0.5, 0.25, and 0.5 µg/mL, respectively) cell lines and inhibited DHFR, TOPO II isomerase, and glutathione reductase (IC_50,_ 2.5, 0.5, and 15.0 µg/mL, respectively) [27]. Panicein F1, a sesquiterpene hydroquinone from *Reniera mucosa* sponge, was found to exhibit DHFRI (IC_50_ 3 µg/mL) (Figure 1) [28].

**Figure 2:**
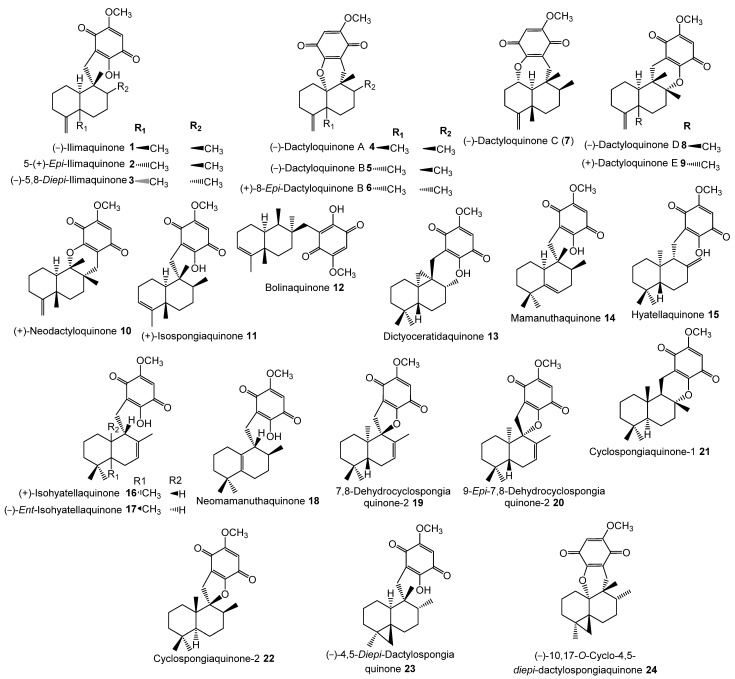
Chemical structures of compounds **1**–**24**.

**Figure 3:**
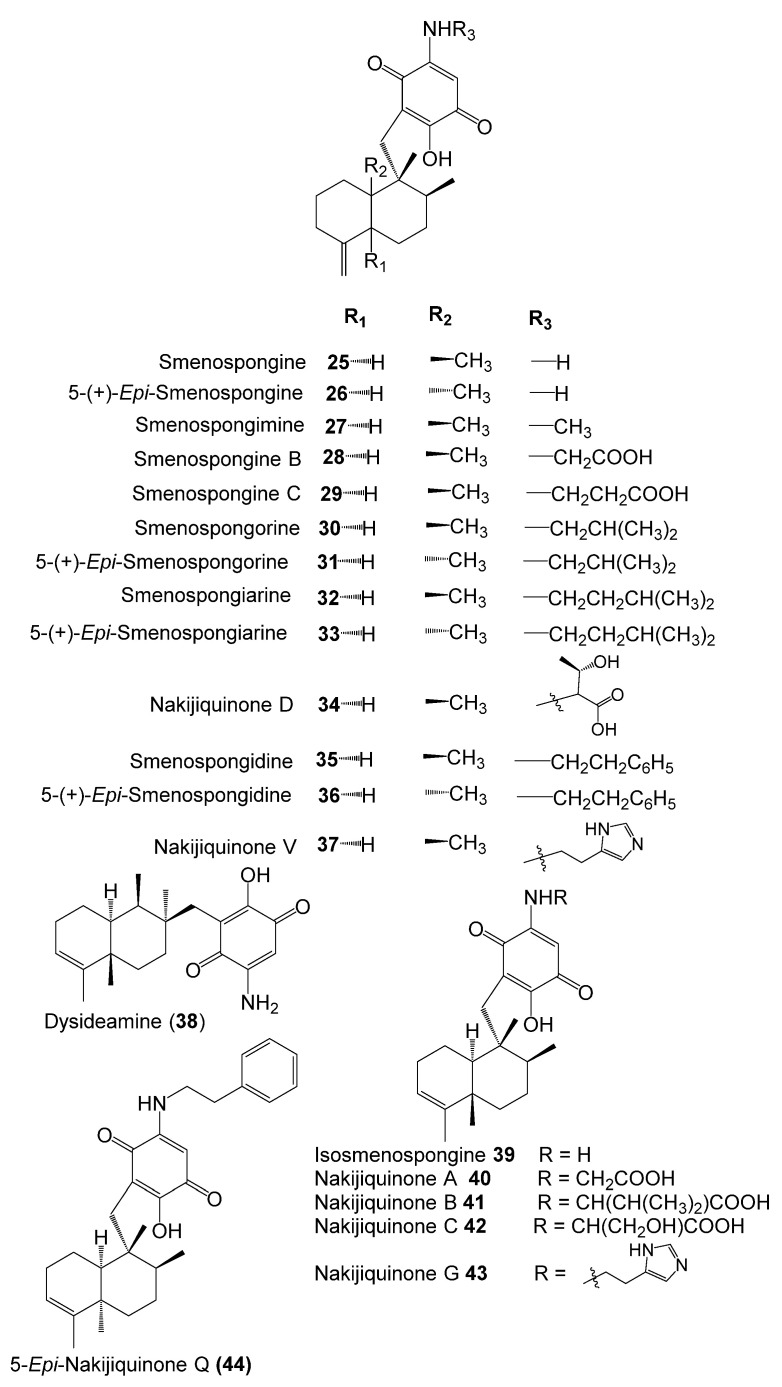
Chemical structures of compounds **25**–**44**.

**Figure 4:**
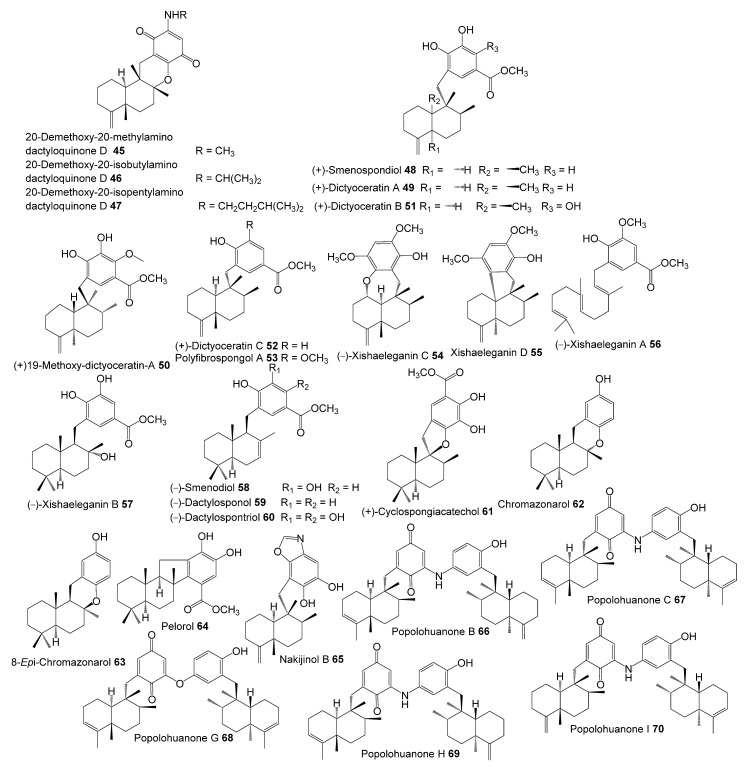
Chemical structures of compounds **45**–**70**.

**Figure 5:**
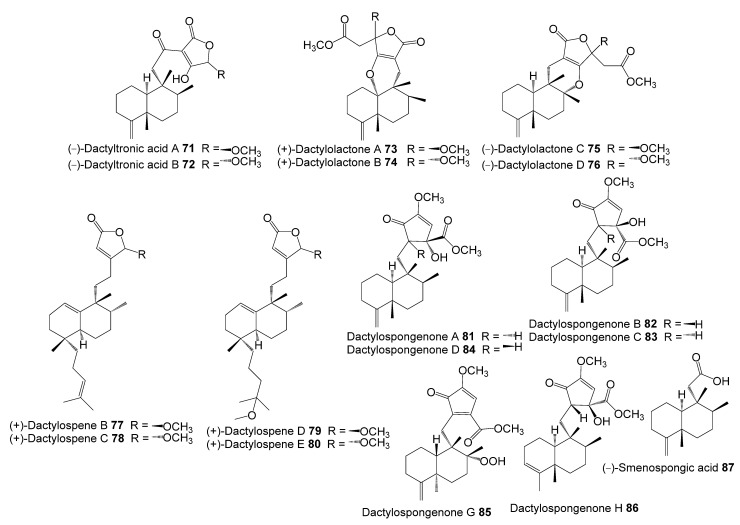
Chemical structures of compounds **71**–**87**.

The inhibitory potential of previously reported quinone-containing terpenoids towards the DHFR suggested that other unexamined and structurally related metabolites, such as sesquiterpenes derived from *D. elegans*, could also have inhibitory effects on the same protein. Therefore, hDHFR was selected as the study target. About 87 metabolites (Figure 2, Figure 3, Figure 4 and Figure 5) were virtually investigated in the docking studies performed entirely in the Schrödinger program (Schrödinger Release 2022-3, LLC, New York, NY, USA, 2021).

It is noteworthy that sesquiterpenes are the prime constituents separated from *D. elegans* that revealed cytotoxic, antitumor, anti-inflammatory, and antibacterial capacities (Table 1) [29].

**Figure 6:**
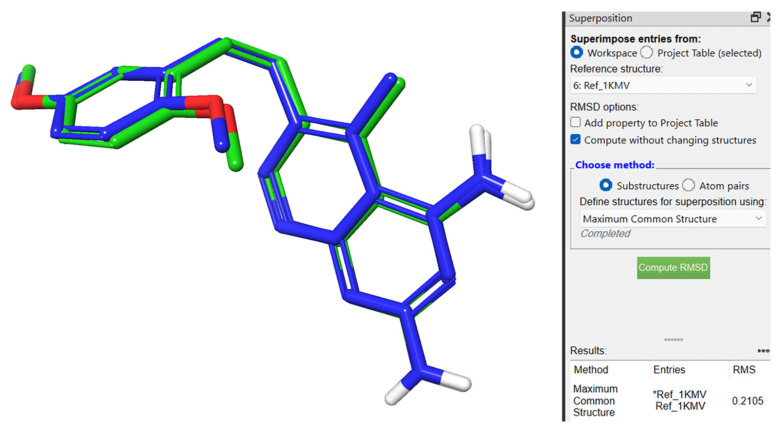
Superimposition of 3D structures with element color atoms of co-crystallized (carbon atoms customized blue) over redocked (carbon atoms customized green) reference inhibitor **SRI-9662** (named Ref_1KMV in the figure).

For validation purposes of the docking method, the co-crystallized inhibitor **SRI-9662** was redocked inside the active site of the prepared hDHFR, and the original and redocked inhibitors were superimposed. By comparing the two results, the redocked inhibitor produced a nearly identical pose to that of the original crystal structure. The calculated root-mean-square deviation (RMSD) value of the superimposition was 0.2105 Å within the acceptable range (Figure 6).

The docking results of the study compounds were listed in Table 2, where they were ranked according to their gscores from highest to lowest in free energy of binding; the more negative scores imply better binding. The top-ranked compounds were **34**, **28,** and **44,** with gscores of −12.431, −11.502, and −10.62 kcal/mol, respectively. The gscores of these compounds exceeded the value of the reference inhibitor **SRI-9662,** which had a binding energy of −10.432 kcal/mol (Table 2). The detailed docking parameters and scores are presented in Table S1.

**Figure 7:**
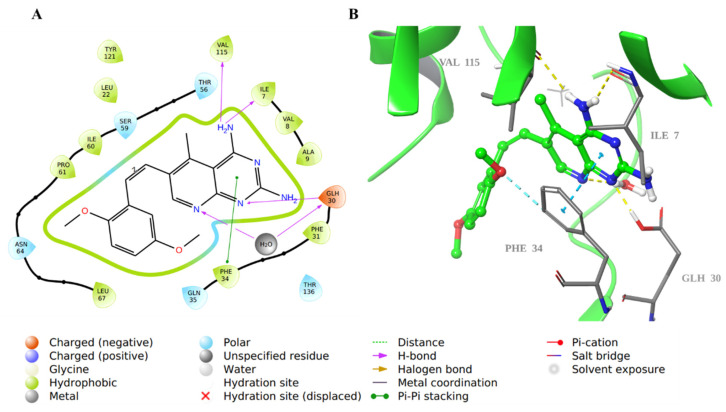
Molecular docking of co-crystallized inhibitor **SRI-9662** in hDHFR (PDB: 1KMV). (**A**) 2D representation of binding interactions of **SRI-9662** with amino acid residues in the active site within a 3 Å distance; (**B**) 3D representation of **SRI-9662** in green within the hDHFR active site. The H-bond and aromatic-hydrogen interactions are in yellow and cyan dotted lines, respectively. A light blue dotted line represents the π-π stacking between aromatic rings.

The 2D and 3D structures of the co-crystallized inhibitor **SRI-9662** revealed that the 4-amino group in the 5-deazapteridine ring interacted with Val115 and Ile7 backbones through hydrogen bonds (H-bond). Moreover, N-1 and N-8 in the ring are also bound through H-bonds to Glu30 with and without a water bridge, respectively. The pyrimidine ring involved in π-π stacking interaction with Phe34, as well as the rest of the molecule, are hydrophobically bound with the nearby hydrophobic residues in the hDHFR active site (Figure 7).

**Figure 8:**
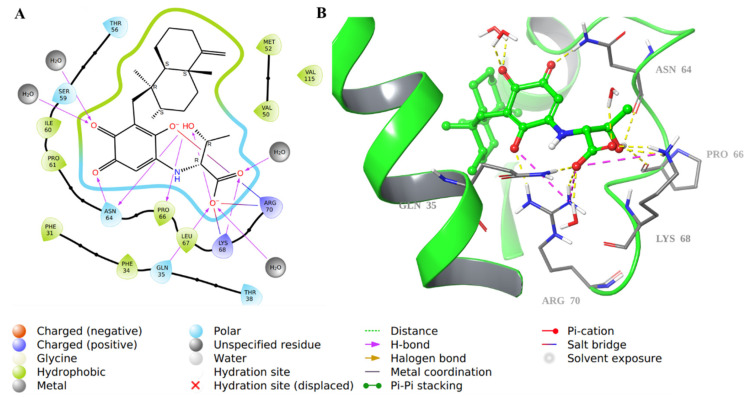
Molecular docking of co-crystallized inhibitor compound **34** in hDHFR (PDB: 1KMV). (**A**) 2D representation of binding interactions of **34** with amino acid residues in the active site within a 3 Å distance; (**B**) 3D representation of **34** in green within the hDHFR active site. The H-bonds and ionic interactions are in yellow and purple dotted lines, respectively.

For compound **34**, there was an array of H-bonding interactions between the carbonyl, hydroxyl, and carboxylic groups in the molecule and amino acid residues such as Asn64, Pro66, Gln35, Lys68, and Arg70. Water molecules were involved as well in such interactions (Figure 8). Additionally, Arg70 formed an ionic bond with the carboxylate ion in **34**, and the decahydronaphthalene ring was involved in hydrophobic interactions.

**Figure 9:**
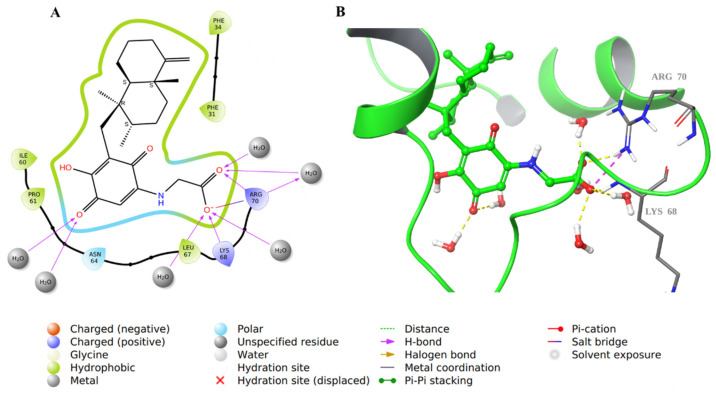
Molecular docking of co-crystallized inhibitor compound **28** in hDHFR (PDB: 1KMV). (**A**) 2D representation of binding interactions of **28** with amino acid residues in the active site within a 3 Å distance; (**B**) 3D representation of **28** in green within the hDHFR active site. The H-bonds and ionic interactions are in yellow and purple dotted lines, respectively.

The structure of compound **28** was very similar to that of compound **34**; hence, it involved similar binding interactions with the active site residues as well as water molecules (Figure 9).

**Figure 10:**
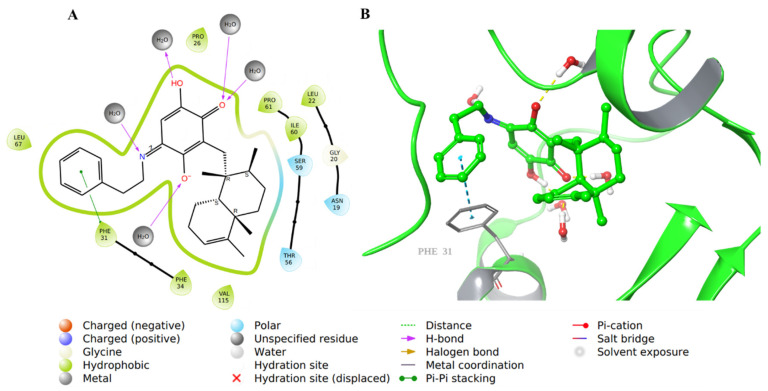
Molecular docking of co-crystallized inhibitor compound **44** in hDHFR (PDB: 1KMV). (**A**) 2D representation of binding interactions of **44** with amino acid residues in the active site within a 3 Å distance; (**B**) 3D representation of **44** in green within the hDHFR active site. The H-bonds and π-π stacking between aromatic rings are in yellow and light blue dotted lines, respectively.

The carboxylic acid was replaced by a phenyl ring that formed a π-π interaction with Phe31 in compound **44** (Figure 10).

### 2.2. In silico ADMET Properties

The Maestro’s QikProp module in Schrödinger was applied to predict the drug-likeness, ADME properties, and toxicity (ADMET) of the metabolites under investigation [30]. Table 3 displayed the ADMET properties and other descriptors. In general, most of the predicted properties for the compounds were within the recommended ranges. However, some compounds fell beyond the recommended ranges of certain descriptors. The logP (QPlogPo/w) and binding to human serum albumin (QPlogKhsa) were high for compounds **66**, **67**, **68**, **69,** and **70**. Additionally, the toxicity was evaluated in terms of the number of reactive functional groups (#rtvFG) and HERG K^+^ channel inhibition (QPlogHERG). It was observed that none of the compounds exceeded the acceptable range (0–2) of reactive groups. However, 11 compounds (**35**, **36**, **37**, **43**, **44**, **56**, **66**, **67**, **68**, **69,** and **70**) were predicted to inhibit the HERG K^+^ channel. The results suggested that the high lipophilicity of these compounds is a factor that contributes to HERG inhibition and plasma protein binding. Moreover, no CNS activity was predicted for any compound. 

### 2.3. MD Simulation

We performed MD simulation for the top three scoring compounds from the docking study (**34**, **28,** and **44**), as well as to the co-crystallized inhibitor **SRI-9662,** using Desmond software in Schrödinger [31,32]. The RMSD of proteins (blue) and ligands (red) are presented at the left and right Y-axes of the plot, respectively.

**Figure 11:**
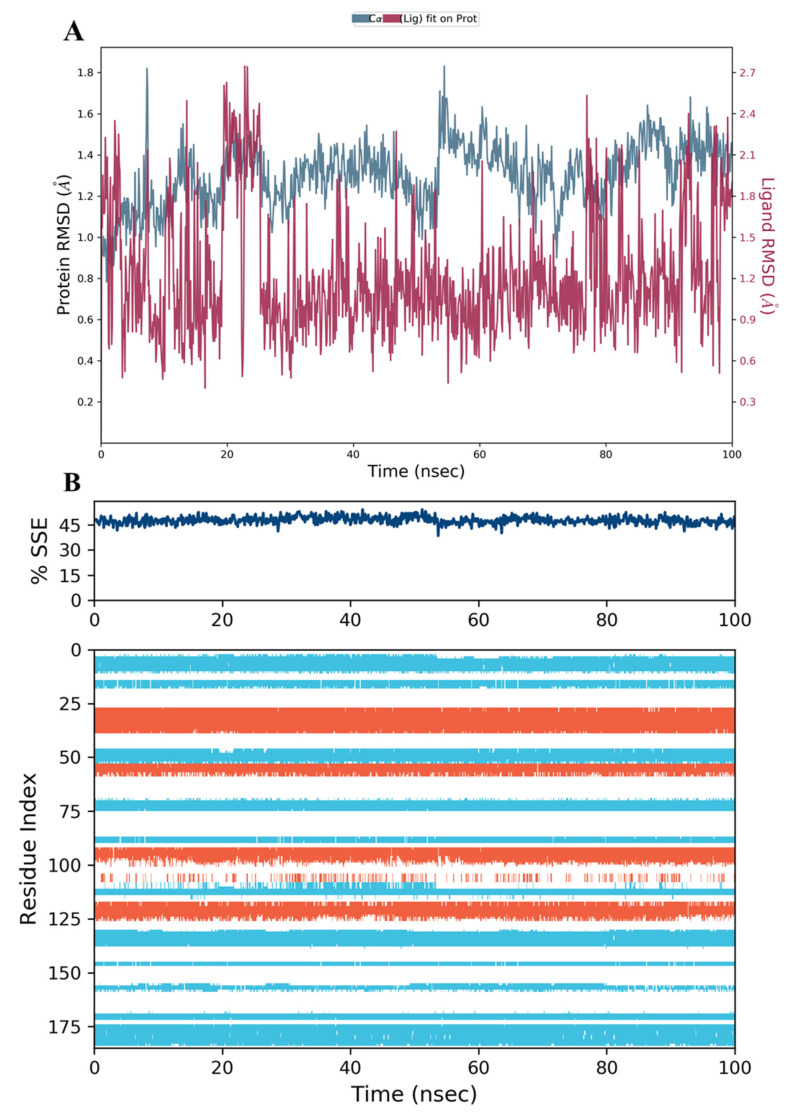
(**A**) The RMSD plot for the reference inhibitor **SRI-9662** complexed with hDHFR (PDB: 1KMV) over a 100 ns simulation time; (**B**) Stability of the hDHFR secondary structure over 100 ns of MD simulation when complexed with **SRI-9662**. Protein secondary structure elements (SSE) like alpha-helices (orange color) and beta-strands (light blue color) were monitored during the simulation. The upper plot reported SSE distribution by residue index across the protein structure, and the plot at the bottom monitored each residue and its SSE assignment over the simulation time.

The results showed that the hDHFR protein and the co-crystallized inhibitor **SRI-9662** were stable during the 100 ns of the simulation run time (Figure 11A). The fluctuations were insignificant and lay within the acceptable range of 1–3 Å (the differences were within 1 and 1.8 Å for the protein and ligand, respectively). This confers a high-binding and stable protein-ligand complex throughout the run.

**Figure 12:**
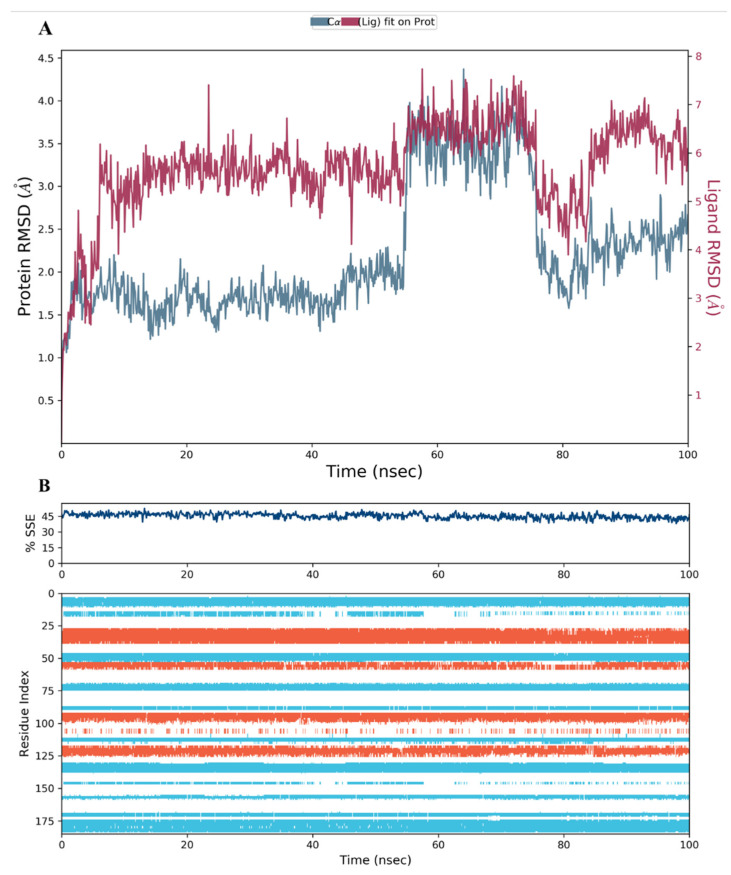
(**A**) The RMSD plot for compound **34** complexed with hDHFR (PDB: 1KMV) over a 100 ns simulation time; (**B**) Stability of the hDHFR secondary structure over 100 ns of MD simulation when complexed with **34**. Protein secondary structure elements (SSE) like alpha-helices (orange color) and beta-strands (light blue color) were monitored during the simulation. The upper plot reported SSE distribution by residue index across the protein structure, and the plot at the bottom monitored each residue and its SSE assignment over the simulation time.

In the case of compounds **34**, the RMSD plot analysis revealed a stable protein-ligand complex, and the fluctuations of both the protein and ligand RMSD charts were within the acceptable range as well (Figure 12A). A small jump in the RMSD of the protein between 55 and 75 ns was observed and then resumed to its normal level until the end of the run. This change was within the acceptable range.

**Figure 13:**
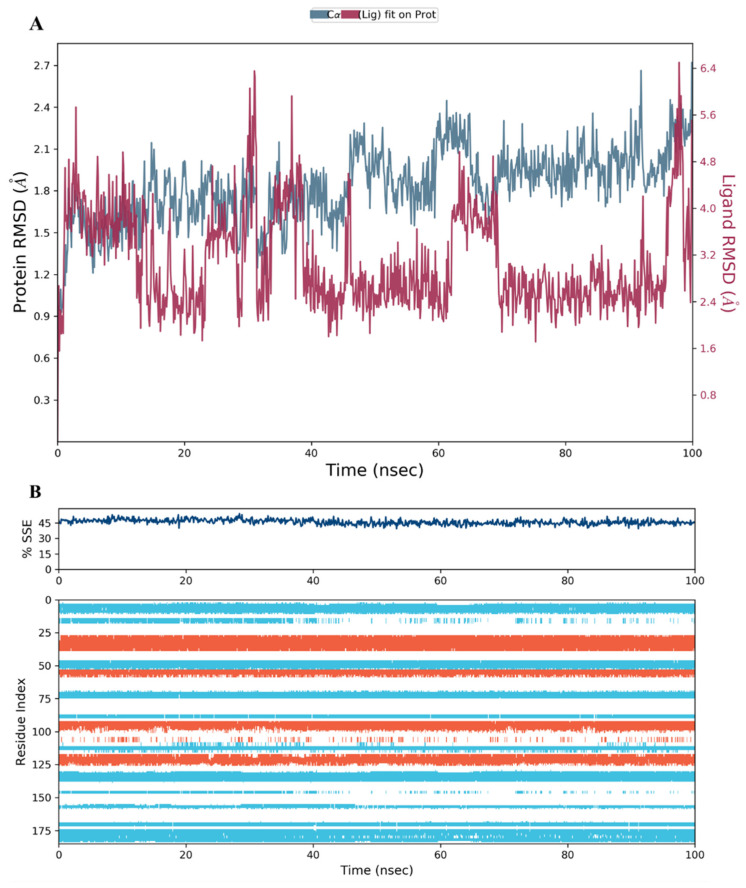
(**A**) The RMSD plot for compound **28** complexed with hDHFR (PDB: 1KMV) over a 100 ns simulation time; (**B**) Stability of the hDHFR secondary structure over 100 ns of MD simulation when complexed with **28**. Protein secondary structure elements (SSE) like alpha-helices (orange color) and beta-strands (light blue color) were monitored during the simulation. The upper plot reported SSE distribution by residue index across the protein structure, and the plot at the bottom monitored each residue and its SSE assignment over the simulation time.

**Figure 14:**
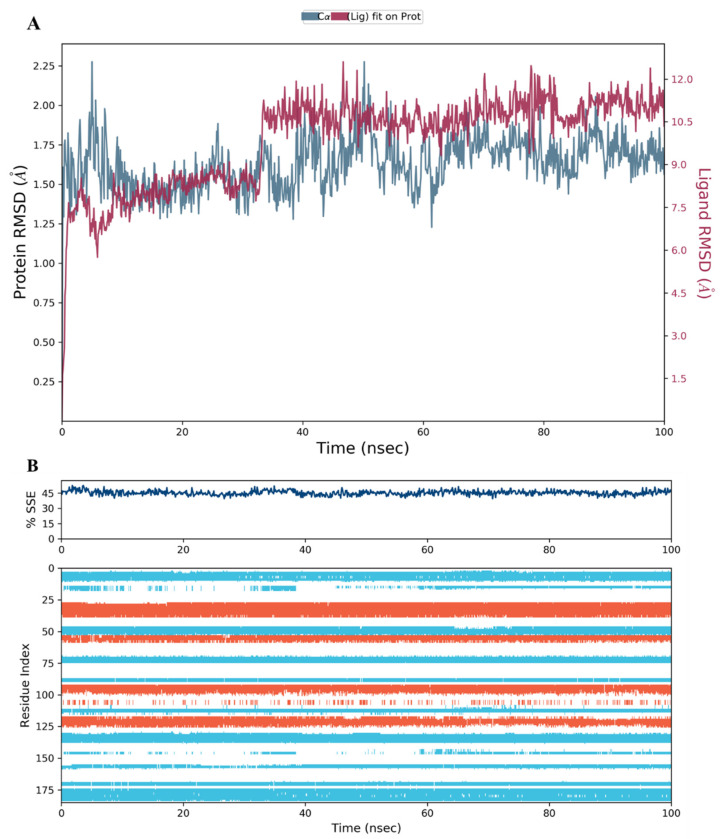
(**A**) The RMSD plot for compound **44** complexed with hDHFR (PDB: 1KMV) over a 100 ns simulation time; (**B**) Stability of the hDHFR secondary structure over 100 ns of MD simulation when complexed with **44**. Protein secondary structure elements (SSE) like alpha-helices (orange color) and beta-strands (light blue color) were monitored during the simulation. The upper plot reported SSE distribution by residue index across the protein structure, and the plot at the bottom monitored each residue and its SSE assignment over the simulation time.

The RMSD of the protein during its interaction with compound **28** was also within range; however, the compound was less stable inside the binding pocket as the RMSD fluctuated slightly beyond the 1–3 Å range (Figure 13A). A possible explanation would be the absence of the 2-hydroxyethyl group, which was the only difference between the **28** and **34** structures (Figure 8A and Figure 9). The chirality of the OH group and adjacent carbon atom may stabilize the carboxylic acid side chain by restricting its free rotation, leading to a decrease in the number of structural conformations available for binding to the protein active site. The RMSD plot analysis of compound **44** was comparable to **34** in terms of complex stability and RMSD fluctuation range (Figure 14A).

The secondary structure elements (SSE), alpha helices in orange and beta strands in light blue, of hDHFR (PDB: 1KMV) complexed with each ligand, were monitored during the 100 ns simulation time. The results showed that the total %SSE was maintained for all ligands and that most of the individual SSEs (by residue index) were stable throughout the run. Minor differences were observed in the turn and loop regions between the reference inhibitor SRI-9662 and the three investigated metabolites (Figure 11B, Figure 12B, Figure 13B, and Figure 14B), as a few parts of these regions were less stable when each of the metabolites interacted with the protein.

**Figure 15:**
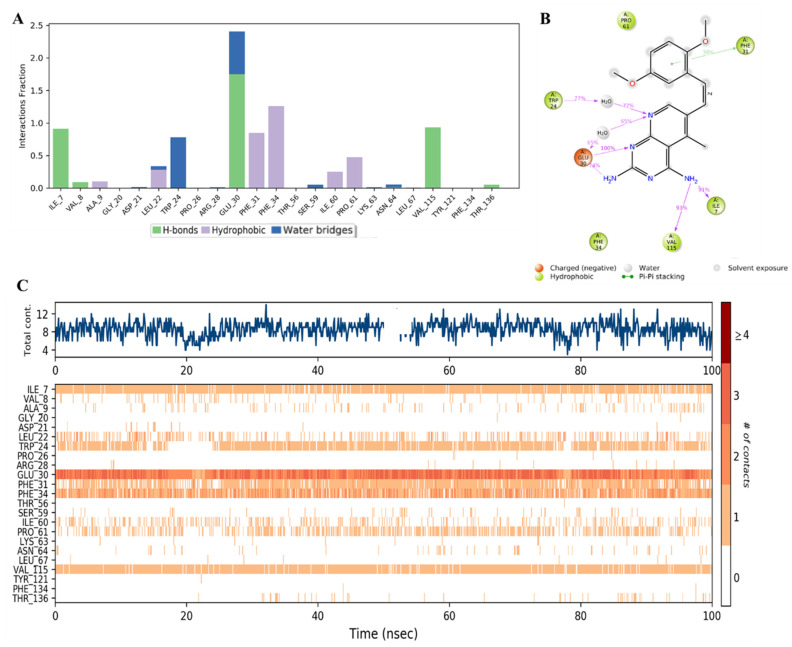
(**A**) Stacked bar graph of hDHFR (PDB: 1KMV) interactions with reference inhibitor **SRI-9662** throughout the simulation. The values were normalized over the course of the trajectory. (**B**) A diagram illustrating the detailed connections of **SRI-9662** with hDHFR. The coordination that appears at least 30% of the designated time period (0.00 to 100.00 nsec) will be presented. It is possible to observe more than 100% as some residues might be linked to the same ligand atom more than once; the pink colored arrows represent the hydrogen bond interaction while the % on the arrows represent the coordination % during the simulation time. (**C**) Timeline of hDHFR- **SRI-9662** interactions presented in (**A**). The top panel presents the total number of specific interactions the protein made with the ligand over the course of the trajectory. The bottom panel presents the residues interacting with the ligand in each trajectory frame. The dark orange color indicates more than one specific interaction is made between some residues and the ligand. As the orange color becomes lighter, the number of contacts between the residue and ligand decreases. The white color indicates no interaction is formed.

Additionally, the MD analysis displayed the specific contact points between the inhibitor and the amino acid residues in the enzyme active site. Figure 15A showed that **SRI-9662** formed strong H-bond interactions with Ile7 and Val115, which lasted about 90% of the simulation time (as demonstrated by the interaction fraction of 0.9).

**Figure 16:**
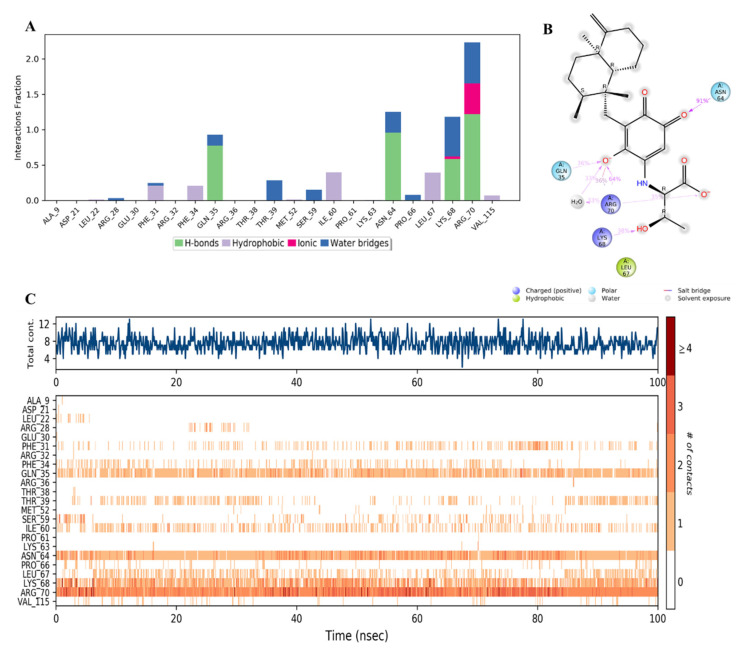
(**A**) Stacked bar graph of hDHFR (PDB: 1KMV) interactions with compound **34** throughout the simulation. The values were normalized over the course of the trajectory. (**B**) A diagram illustrating the detailed connections of **34** with hDHFR. The coordination that appears at least 30% of the designated time period (0.00 to 100.00 nsec) will be presented. It is possible to observe more than 100% as some residues might be linked to the same ligand atom more than once; the pink colored arrows represent the hydrogen bond interaction while the % on the arrows represent the coordination % during the simulation time. (**C**) Timeline of hDRFR-**34** interactions presented in **A**. The top panel presents the total number of specific interactions the protein made with the ligand over the course of the trajectory. The bottom panel presents the residues interacting with the ligand in each trajectory frame. The dark orange color indicates more than one specific interaction is made between some residues and the ligand. As the orange color becomes lighter, the number of contacts between the residue and ligand decreases. The white color indicates no interaction is formed.

However, the strongest binding interaction was observed with Glu30, which involved multiple H-bonds with the 2-amino, N-1, and N-8 of the 5-deazapteridine ring either directly or through a water bridge (Figure 15B). The combined effect of these interactions was maintained for about 240% of the run time (Figure 15A). An H-bond through a water bridge was also formed with Trp24. Additional significant hydrophobic interactions with values near or above 1 were observed with Phe31 and Phe34, as well as, to a lesser extent, with Pro61. Detailed ligand atom interactions that lasted more than 30% of the simulation time with selected amino acid residues are presented in Figure 16B. The dimethoxyphenyl ring formed a π-π stacking interaction with Phe31, and the amino groups and nitrogen atoms of the 5-deazapteridine ring H-bonded with several residues, as detailed above. Moreover, the total specific interactions were presented in the top panel of Figure 16C, while the bottom plot showed the ligand-protein interactions by residues in each trajectory frame. The darker and more continuous the orange color is, the stronger the binding, which was observed with the key residues of Ile7, Trp24, Glu30, Phe31, Phe34, and Val115.

It was observed that compound **34** was bound to a different set of amino acid residues in the active site of hDHFR compared to the native inhibitor. The differences could be related to the polar nature of **34**. The major types of interactions between **34** and the active residues were the direct and water-aided H-bonds with Gln35, Asn64, Lys68, and Arg70, where the latter was the strongest (> 200%) and involved in additional ionic interaction with the carboxylate anion in the ligand (Figure 16A–C). Minor hydrophobic contacts were formed with Phe31, Phe34, Ile60, and Leu67 with <50% (Figure 16A).

**Figure 17:**
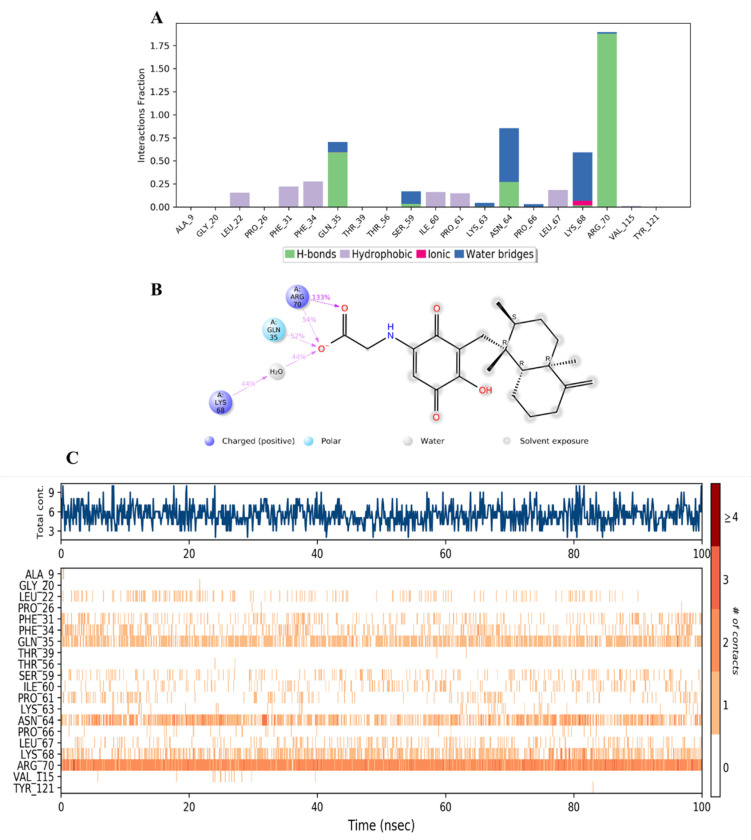
(**A**) Stacked bar graph of hDHFR (PDB: 1KMV) interactions with compound **28** throughout the simulation. The values were normalized over the course of the trajectory. (**B**) A diagram illustrating the detailed connections of **28** with hDHFR. The coordination that appears at least 30% of the designated time period (0.00 to 100.00 nsec) will be presented. It is possible to observe more than 100% as some residues might be linked to the same ligand atom more than once; the pink colored arrows represent the hydrogen bond interaction while the % on the arrows represent the coordination % during the simulation time. (**C**) Timeline of hDRFR-**28** interactions presented in (**A**). The top panel presents the total number of specific interactions the protein made with the ligand over the course of the trajectory. The bottom panel presents the residues interacting with the ligand in each trajectory frame. The dark orange color indicates more than one specific interaction is made between some residues and the ligand. As the orange color becomes lighter, the number of contacts between the residue and ligand decreases. The white color indicates no interaction is formed.

Similar binding modes were observed with compound **28** and the key residues mentioned above (Figure 17A–C), owing to the structural similarity between **28** and **34**.

**Figure 18:**
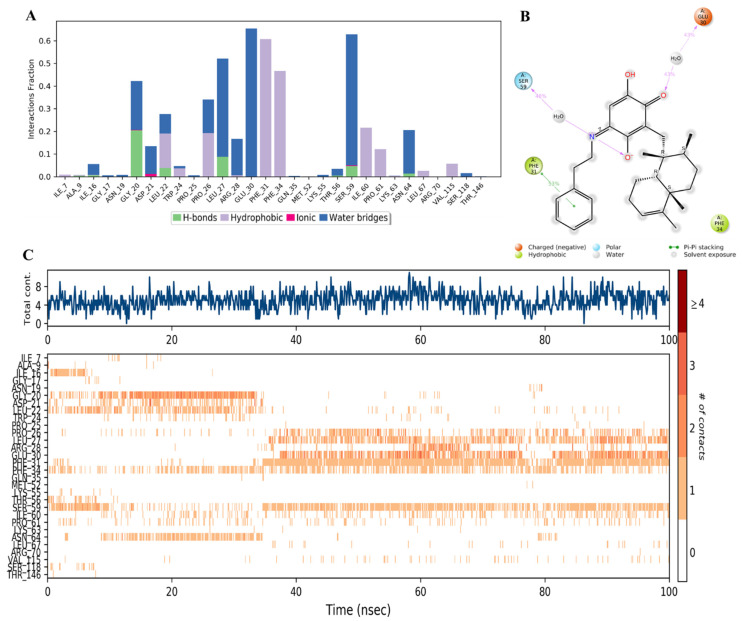
(**A**) Stacked bar graph of hDHFR (PDB: 1KMV) interactions with compound **44** throughout the simulation. The values were normalized over the course of the trajectory. (**B**) A diagram illustrating the detailed connections of **44** with hDHFR. The coordination that appears at least 30% of the designated time period (0.00 to 100.00 nsec) will be presented. It is possible to observe more than 100% as some residues might be linked to the same ligand atom more than once; the pink colored arrows represent the hydrogen bond interaction while the % on the arrows represent the coordination % during the simulation time. (**C**) Timeline of hDRFR-**44** interactions is presented in (**A**). The top panel presents the total number of specific interactions the protein made with the ligand over the course of the trajectory. The bottom panel presents the residues interacting with the ligand in each trajectory frame. The dark orange color indicates more than one specific interaction is made between some residues and the ligand. As the orange color becomes lighter, the number of contacts between the residue and ligand decreases. The white color indicates no interaction is formed.

Moreover, it was found that compound **44** bound to amino acid residues of Phe31, Phe34, and Glu30 in the active site (Figure 18A) similar to the reference inhibitor (Figure 16A). It might be due to the hydrophobic nature of **44**, in which the phenyl ring in the side chain replaced the carboxylic acid in compounds **34** and **28,** which formed strong hydrogen and ionic interactions with the protein. Compound **44** formed hydrophobic interactions with Phe31 and Phe34 and H-bonds mostly in the presence of water molecules with Gly20, Leu27, Glu30, and Ser59 (Figure 18A,B). However, the availability of these interactions did not exceed 60% of the simulation time. The inconsistent binding affinity was also demonstrated by the low total number of specific contacts and incomplete orange lines with each residue in Figure 18C, top and bottom panels, respectively.

## 3. Materials and Methods

### 3.1. Ligand and Protein Preparation

The docking study was performed with the Schrodinger program (Schrödinger Release 2022-3: Schrödinger, LLC, New York, NY, USA, 2021). The crystal structure of hDHFR complexed with the **SRI-9662** inhibitor was downloaded from the protein data bank (PDB; ID: 1KMV) [33]. The protein was prepared using the “Protein Preparation Wizard” tool in Maestro software, where the missing hydrogens were added to the residues, the metal ionization state was corrected, and the water molecules > 5 Å from protein residues were deleted. The protein was then refined by predicting the pKa of the ionizable residues using PROPKA and water molecules >3 Å (not involved in the water bridge), which were removed [34]. Finally, the protein was minimized by applying the OPLS4 force field. All ligands (sesquiterpene metabolites in addition to the co-crystallized inhibitor **SRI-9662**) were prepared before docking using the “LigPrep” tool, where their 2D structures were converted to 3D and energy-minimized using the OPLS3 force field [35]. The hydrogens were added, and all possible ionization states and tautomeric forms were created at a pH of 7.0 ± 0.2 by Epik; a desalt option was also chosen. The H-bonds were optimized by predicting the pKa of ionizable groups using PROPKA [34].

### 3.2. Grid Generation and Molecular Docking

Before docking, a grid box was generated around the active site of hDHFR (PDB: 1KMV) containing the co-crystallized inhibitor **SRI-9662,** aided by Glide’s “Receptor-Grid-Generation” tool in the Schrödinger suite [36]. The box was built around the co-crystallized ligand by selecting the “centroid of workspace ligand” function. The length of the box in each of the X, Y, and Z dimensions was set by default at 10 Å. All compounds under investigation were docked inside the grid box once with standard precision (SP) and three times with extra precision (XP) protocols (for validation purposes), and all other parameters were set to default [37]. The non-polar atoms were set for the VdW radii scaling factor at 1.0, and the partial charge cutoff was 0.25. Finally, the “Ligand Docking” tool was implemented for docking [38]. To further validate the docking method, the co-crystallized inhibitor was re-docked inside the grid box and evaluated. The docking results were assessed in terms of the gscore (ranks different compounds), emodel (ranks different conformers), and XP gscore. Glide uses emodel scoring to select the best poses of the docked compounds; then, it ranks the best poses based on the given gscores. The XP gscore ranks the poses generated by the XP Glide mode. The XP Glide takes into consideration the major driving forces and structural motifs that contributed to protein-ligand binding affinity, as described in the following equations [37]:

XP Glide Score = *E*_coul_ + *E*_vdW_ + *E*_bind_ + *E*_penalty_

*E*_bind_ = *E*_hyd_enclosure_ + *E*_hb_nn_motif_ + *E*_hb_cc_motif_ + *E*_PI_ + *E*_hb_pair_ + *E*_phobic_pair_

*E*_penalty_ = *E*_desolv_ + *E*_ligand_strain_

where:

*E* is energy (calculated for each of the following descriptors); *E*_coul_ is Coulomb energy, *E*_vdW_ is van der Waal, *E*_bind_ is the energy that favors binding, *E*_penalty_ is the penalty that disfavors binding, *E*_hyd_enclosure_ is hydrophobic enclosure, *E*_hb_nn_motif_ is special neutral-neutral hydrgen-bond motifs, *E*_hb_cc_motif_ is special charged-charged hydrogen-bond motifs, *E*_PI_ is pi-cation interactions, *E*_hb_pair_ is hydrogen bond pair, *E*_phobic_pair_ is lipophilic pair, and *E*_desolv_ is desolvation energy [37].

The Molecular Mechanics Generalized Born Surface Area (MMGBSA) method was used to calculate the binding free energy (Δ*G*) of the protein-ligand complexes in a solvent based on the following equation:

Δ*G* _binding_ = *G* _complex_ − (*G* protein + *G* _ligand_)

where *G* _complex_ is the free energy of the protein-ligand complex, *G* _protein_ and *G* _ligand_ are the free energies of unbound protein and ligand in the solvent, respectively [39,40].

### 3.3. ADME Properties

The ADME properties (absorption, distribution, metabolism, and excretion) and toxicity of compounds under investigation were predicted using the QikProp module of the Schrodinger suite [30]. This module is useful in predicting the physicochemical properties and other descriptors to facilitate the drug discovery and development process by identifying and eliminating non-drug-like compounds from entering the clinical stage and failing. The predicted descriptors were molecular weight (mol_MW), drug-likeness (#Stars), total solvent accessible surface area (SASA), number of hydrogen bond donors and acceptors (donorHB and acceptHB), predicted octanol-water partitioning (QPlogPo/w), estimated binding to human serum albumin (QPlogKhsa), number of the possible metabolites (# metab), predicted blood-brain partitioning (QPlogBB), percentage of human oral absorption, predicted IC_50_ for inhibiting HERG-K^+^ channels (QPogHERG), predicted apparent Caco-2 cell permeability in nm/s for gut-blood barrier (QPPCaco), central nervous system activity (CNS), number of reactive functional groups present (#rtvFG), and percent of human oral absorption. The predicted values were then compared to the recommended range derived from values determined for 95% of known drugs.

### 3.4. MD Simulation

MD simulations were performed using Desmond in the Schrödinger package [31,32]. The software created a simulated environment that resembles the dynamic nature of the molecular system under physiological conditions to evaluate the virtual stability of interactions between the protein and ligand [41]. The RMSD plots assess the stability of protein-ligand complexes by calculating the deviation of the protein and ligand atoms inside the binding pocket at the end of the simulation time of 100 ns and comparing the results to their initial positions before the simulation at 0 ns [42]. The hDHFR protein was first complexed with the desired ligand in the docking experiment. The protein-ligand complex was then tuned through the “System-Builder” tool to generate the solvated system for simulation. The solvent model was set to TIP3P, the selected box shape was orthorhombic, and the box dimensions were 10 Å. Sodium ions (Na^+^) were added to neutralize the system. The simulation parameters were set up in the “Molecular Dynamic” tool, where the protein-ligand complex was evaluated at pH 7.0 ± 0.2 over a simulation time of 100 ns. The ensemble class was set as NPT to maintain a constant temperature and pressure of 300 K and 1.01325 bar, respectively, throughout the run. The generated results were analyzed at the end of the MD simulation.

## 4. Conclusion

Our findings suggested that **34**, **28**, and **44** possessed potent interactions with the active site of hDHFR compared to **SRI-9662** (reference standard), as demonstrated by the docking studies. The MD simulation revealed detailed information about protein-ligand complex stability and specific binding contacts between each ligand and the hDHFR binding site. Additionally, the ADMET prediction demonstrated that all physicochemical parameters and ADMET properties are within the satisfactory range described for human treatment for the majority of sesquiterpene metabolites. These metabolites could be possible leads for DHFRI candidates; however, more in vitro, in vivo, and mechanistic investigation, as well as developing semi- and synthetic derivatives to enhance their DHFRI effectiveness, should be the focus of future research.

## Figures and Tables

**Table 1:** Reported promising bio-activities of *Dactylospongia elegans* sesquiterpenes [29].

Activity	Compound Name
Cytotoxicity	(−)-Ilimaquinone (**1**), 5-(+)-*epi*-ilimaquinone (**2**), (−)-dactyloquinone B (**5**), (+)-isospongiaquinone (**11**), mamanuthaquinone (**14**), hyatellaquinone (**15**), (+)-isohyatellaquinone (**16**), neomamanuthaquinone (**18**), 9-*epi*-7,8-dehydrocyclospongiaquinone-2 (**20**), smenospongine (**25**), smenospongimine (**27**), smenospongine b (**28**), smenospongine c (**29**), smenospongorine (**30**), smenospongiarine (**32**), 5-(+)-*epi*-smenospongiarine (**33**), smenospongidine (**35**), 5-(+)-*epi*-smenospongidine (**36**), isosmenospongine (**39**), nakijiquinone A (**40**), nakijiquinone B (**41**), nakijiquinone G (**43**), 5-*epi*-nakijiquinone Q (**44**), (+)-dictyoceratin A (**49**), (+)-19-methoxy-dictyoceratin-A (**50**), (+)-dictyoceratin B (**51**), (+)-dictyoceratin C (**52**), nakijinol B (**65**), (+)-dactylospene C (**78),** dactylospongenone A (**81**)
Antibacterial	(−)-ilimaquinone (**1**), 5-(+)-*epi*-ilimaquinone (**2**), (−)-dactyloquinone A (**4**), (−)-dactyloquinone B (**5**), (−)-dactyloquinone C (**7**), (−)-dactyloquinone D (**8**), (+)-dactyloquinone E (**9**), (+)-isospongiaquinone (**11**), smenospongine C (**29**), smenospongorine (**30**), 5-(+)-*epi*-smenospongidine (**36**), isosmenospongine (**39**), nakijiquinone A (**40**), nakijiquinone B (**41**), 5-*epi*-nakijiquinone Q (**44**), (+)-dictyoceratin A (**49**), (+)-dictyoceratin B (**51**), (−)-xishaeleganin C (**54**), (+)-xishaeleganin d (**55**), (−)-xishaeleganin B (**57**), pelorol (**64**), (−)-dactyltronic acid A (**71**), (−)-dactyltronic acid B (**72**),
Anti-inflammatory	(+)-dactylospene B (**77**), (+)-dactylospene C (**78)**
Antitrypanosomal	(−)-ilimaquinone (**1**), pelorol (**64**)
Antimalarial	(−)-ilimaquinone (**1**), pelorol (**64**)
*β*-Secretase 1 inhibition	(−)-ilimaquinone (1), smenospongine (**25**)

**Table 2:** In silico docking results of sesquiterpene metabolites with hDHFR (PDB: 1KMV) compared to the reference inhibitor **SRI-9662**.

Compounds	PubChem CID/ChemSpider ID	XP gscore	glide gscore	Prime Energy
**34**	11744241	−12.431	−12.431	−7441.3
**28**	50994611	−11.502	−11.502	−7452.5
**44**	31130045 *	−10.62	−10.62	−7460.7
**Ref_ SRI-9662**		−10.432	−10.432	−7788.8
**51**	21681043	−9.874	−9.874	−7489.4
**61**	71726095	−9.71	−9.71	−7448.6
**50**	-	−9.598	−9.598	−7483.7
**84**	10369050 *	−9.341	−9.341	−7458.4
**65**	50994610	−8.932	−8.932	−7475
**36**	-	−8.884	−8.884	−7459.1
**30**	101245402	−8.755	−8.755	−7453.3
**15**	10361056	−8.705	−8.705	−7455.7
**3**	30770979 *	−8.592	−8.592	−7465.9
**14**	495021	−8.494	−8.494	−7475.6
**52**	21589795	−8.42	−8.42	−7522.7
**41**	457734	−8.407	−8.407	−7399.1
**85**	64063250 *	−8.358	−8.358	−7467.9
**56**	-	−8.288	−8.288	−7546.5
**2**	21727418	−8.256	−8.256	−7465.1
**42**	10071409	−8.154	−8.154	−7385.6
**18**	24691897	−7.953	−7.953	−7447.5
**82**	132498497	−7.949	−7.949	−7431.7
**80**	-	−7.881	−7.881	−7569.4
**64**	10067895	−7.876	−7.876	−7478.7
**60**	10316629 *	−7.867	−7.867	−7472.2
**16**	24905924	−7.836	−7.836	−7444.7
**55**	-	−7.825	−7.825	−7459.6
**35**	14286425	−7.816	−7.816	−7435.1
**17**	27023531	−7.794	−7.794	−7421.4
**13**	14526059	−7.711	−7.711	−7477.8
**31**	-	−7.711	−7.711	−7426.8
**43**	24710044 *	−7.59	−7.59	−7373.1
**57**	-	−7.58	−7.58	−7511.6
**48**	101605919	−7.56	−7.56	−7499.8
**49**	9885835	−7.56	−7.56	−7499.8
**1**	72291	−7.5	−7.5	−7460.7
**40**	457733	−7.368	−7.368	−7374.2
**63**	102015226	−7.335	−7.335	−7514
**38**	44188455	−7.31	−7.31	−7446.5
**39**	-	−7.211	−7.211	−7435.4
**33**	21727419	−7.208	−7.208	−7445.6
**23**	-	−7.147	−7.147	−7460.2
**62**	189164	−7.136	−7.136	−7520.3
**79**	-	−7.116	−7.116	−7556.1
**22**	23424798	−7.071	−7.071	−7443
**21**	25211413	−7.062	−7.062	−7463.5
**6**	-	−6.942	−6.942	−7423.3
**83**	132498496	−6.719	−6.719	−7459.3
**25**	3081931	−6.583	−6.583	−7467.6
**26**	10617363	−6.567	−6.567	−7469.5
**77**	-	−6.54	−6.54	−7545.1
**78**	-	−6.316	−6.316	−7553.1
**76**	11811553	−6.303	−6.303	−7523.8
**54**	-	−6.274	−6.274	−7479.5
**5**	10915278	−6.194	−6.194	−7435.8
**53**	9977451	−6.145	−6.145	−7486.6
**9**	12972982	−6.101	−6.101	−7439
**70**	65790999 *	−6.055	−6.055	−7459.8
**29**	50994612	−5.927	−5.927	−7442
**32**	10313302 *	−5.704	−5.704	−7450.8
**4**	11035675	−5.579	−5.579	−7423.7
**20**	27023533 *	−5.371	−5.371	−7440.4
**24**	-	−5.314	−5.314	−7422.7
**75**	11090757	−5.228	−5.228	−7534.9
**7**	12972980	−5.134	−5.134	−7462.2
**81**	102284910	−5.034	−5.034	−7436.4
**10**	637868	−4.462	−4.462	−7442.6
**37**	73930387 *	−4.239	−4.239	−7446.9
**45**	132606991	−3.689	−3.689	−7417.7
**12**	10066979	−3.676	−3.676	−7363
**46**	132606990	−3.433	−3.433	−7414.9
**47**	107805883 *	−3.308	−3.308	−7429.9
**27**	132578684	−3.139	−3.139	−7406.7
**8**	12972981	−3.13	−3.13	−7439.4
**58**	10316627 *	−2.619	−2.619	−7455.7
**73**	10873154	−2.199	−2.199	−7530
**74**	11014966	−0.852	−0.852	−7471.3
**72**	-	−0.122	−0.122	−7578.1
**71**	54729714	0.84	0.84	−7579.9

* ChemSpider ID.

**Table 3:** ADMET prediction of the tested sesquiterpenes using QickProp.

Molecule	mol_MW	#Stars	#rtvFG	CNS	SASA	donorHB	accptHB	QPlogPo/w	QPlogHERG	QPPCaco	QPlogBB	#metab	QPlogKhsa	Percent HumanOral Absorption
Recommended Range	(130–725)	(0.0–5.0)	(0–2)	(−2 Inactive) (+2 Active)	(300–1000)	(0–6)	(2.0–20.0)	(−2–6.5)	Concern Below −5	<25 Poor, >500 Great	(−3–1.2)	(1–8)	(−1.5–1.5)	(<25% Poor; >80% High)
**1**	358.48	0.00	2.00	0.00	590.81	1.00	5.50	3.46	−3.66	1182.68	−0.51	4.00	0.46	100.00
**2**	358.48	0.00	2.00	0.00	587.02	1.00	5.50	3.40	−3.59	1076.04	−0.54	4.00	0.45	100.00
**3**	358.48	0.00	2.00	0.00	603.35	1.00	5.50	3.45	−3.87	902.98	−0.64	4.00	0.49	100.00
**4**	356.46	1.00	0.00	0.00	573.09	0.00	5.50	3.29	−3.50	1942.90	−0.12	3.00	0.27	100.00
**5**	356.46	1.00	0.00	0.00	567.60	0.00	5.50	3.23	−3.38	1659.36	−0.17	3.00	0.27	100.00
**6**	356.46	1.00	0.00	0.00	575.08	0.00	5.50	3.26	−3.56	1778.31	−0.16	3.00	0.27	100.00
**7**	356.46	1.00	0.00	0.00	570.26	0.00	5.50	3.26	−3.50	1751.98	−0.15	3.00	0.27	100.00
**8**	356.46	1.00	0.00	0.00	582.17	0.00	5.50	3.30	−3.66	1521.91	−0.22	3.00	0.31	100.00
**9**	356.46	1.00	0.00	0.00	567.44	0.00	5.50	3.24	−3.32	1484.16	−0.20	3.00	0.29	100.00
**10**	356.46	1.00	0.00	0.00	587.87	0.00	5.50	3.29	−3.78	1451.01	−0.26	3.00	0.31	100.00
**11**	358.48	0.00	2.00	0.00	593.80	1.00	5.50	3.46	−3.75	1173.47	−0.52	5.00	0.46	100.00
**12**	358.48	0.00	2.00	−1.00	621.90	1.00	5.50	3.47	−4.21	737.72	−0.77	5.00	0.52	100.00
**13**	358.48	0.00	2.00	−1.00	609.27	1.00	5.50	3.39	−3.85	768.06	−0.72	3.00	0.50	100.00
**14**	358.48	0.00	2.00	0.00	604.81	1.00	5.50	3.53	−3.78	1069.35	−0.57	5.00	0.51	100.00
**15**	358.48	0.00	2.00	−1.00	620.06	1.00	5.50	3.45	−4.10	701.23	−0.78	5.00	0.52	100.00
**16**	358.48	0.00	2.00	−1.00	607.51	1.00	5.50	3.43	−3.91	760.42	−0.71	6.00	0.50	100.00
**17**	358.48	0.00	2.00	−1.00	607.82	1.00	5.50	3.42	−3.93	749.67	−0.72	6.00	0.50	100.00
**18**	344.45	0.00	2.00	−1.00	613.23	1.00	5.50	3.30	−4.02	715.47	−0.77	6.00	0.46	100.00
**19**	356.46	1.00	0.00	0.00	598.49	0.00	5.50	3.34	−3.94	1279.73	−0.32	4.00	0.35	100.00
**20**	356.46	1.00	0.00	0.00	598.60	0.00	5.50	3.38	−3.90	1348.28	−0.30	4.00	0.36	100.00
**21**	358.48	1.00	0.00	0.00	595.10	0.00	5.50	3.30	−3.72	1200.63	−0.34	2.00	0.35	100.00
**22**	358.48	1.00	0.00	0.00	596.11	0.00	5.50	3.33	−3.74	1332.29	−0.30	2.00	0.36	100.00
**23**	358.48	0.00	2.00	0.00	603.69	1.00	5.50	3.40	−3.76	861.92	−0.66	3.00	0.49	100.00
**24**	356.46	1.00	0.00	0.00	572.49	0.00	5.50	3.24	−3.41	1899.93	−0.13	2.00	0.27	100.00
**25**	343.47	0.00	2.00	−1.00	569.45	2.50	5.75	2.50	−3.62	347.02	−0.96	4.00	0.27	87.04
**26**	343.47	0.00	2.00	−1.00	561.62	2.50	5.75	2.43	−3.46	314.54	−0.98	4.00	0.26	85.85
**27**	357.49	0.00	2.00	0.00	605.48	2.00	5.75	3.23	−3.86	834.01	−0.67	5.00	0.44	100.00
**28**	401.50	0.00	0.00	−2.00	647.79	3.00	7.75	2.60	−2.32	23.48	−1.76	6.00	0.02	66.70
**29**	415.53	0.00	2.00	−2.00	687.22	2.00	6.75	3.68	−2.66	24.51	−1.88	6.00	0.34	73.34
**30**	399.57	0.00	0.00	−1.00	697.89	2.00	4.50	4.94	−4.54	1304.45	−0.75	4.00	0.97	100.00
**31**	399.57	0.00	0.00	−1.00	665.46	2.00	4.50	4.80	−4.02	1148.84	−0.74	4.00	0.93	100.00
**32**	413.60	0.00	0.00	−1.00	724.46	2.00	4.50	5.26	−4.69	1304.85	−0.83	4.00	1.06	100.00
**32**	413.60	2.00	0.00	−1.00	732.98	2.00	4.50	5.27	−4.80	1146.67	−0.91	4.00	1.08	100.00
**33**	413.60	0.00	0.00	−1.00	712.20	2.00	4.50	5.25	−4.54	1450.92	−0.76	4.00	1.05	100.00
**34**	445.56	0.00	0.00	−2.00	649.73	3.00	7.20	3.39	−1.74	27.35	−1.73	6.00	0.22	72.53
**35**	447.62	0.00	2.00	−1.00	750.39	1.00	7.00	4.95	−5.55	1120.27	−0.79	5.00	0.88	100.00
**36**	447.62	0.00	2.00	−1.00	738.69	1.00	7.00	4.90	−5.31	959.48	−0.82	5.00	0.89	100.00
**37**	437.58	0.00	2.00	−2.00	733.50	3.00	7.25	3.79	−5.31	363.49	−1.37	6.00	0.58	94.97
**38**	343.47	0.00	2.00	−2.00	597.78	2.50	5.75	2.48	−4.13	216.02	−1.25	5.00	0.30	83.25
**39**	343.47	0.00	2.00	−1.00	569.70	2.50	5.75	2.49	−3.65	343.89	−0.97	5.00	0.26	86.93
**40**	401.50	0.00	0.00	−2.00	648.04	3.00	7.75	2.60	−2.35	23.48	−1.76	7.00	0.02	66.69
**41**	443.58	1.00	0.00	−2.00	712.86	3.00	7.75	3.79	−2.54	58.16	−1.49	7.00	0.34	80.71
**42**	431.53	0.00	2.00	−2.00	673.74	3.00	8.45	2.52	−2.39	11.60	−2.19	8.00	−0.02	60.74
**43**	437.58	0.00	2.00	−2.00	728.41	3.00	7.25	3.60	−5.16	254.87	−1.52	7.00	0.56	91.10
**44**	447.62	1.00	2.00	−1.00	760.65	1.00	7.00	4.89	−5.82	772.87	−0.98	6.00	0.89	100.00
**45**	355.48	0.00	0.00	0.00	605.15	1.00	4.50	3.86	−4.03	1096.57	−0.46	3.00	0.74	100.00
**46**	397.56	1.00	0.00	0.00	695.14	1.00	4.50	5.06	−4.59	1512.13	−0.51	3.00	1.12	100.00
**47**	411.58	1.00	0.00	0.00	726.02	1.00	4.50	5.47	−4.86	1727.51	−0.54	3.00	1.23	100.00
**48**	372.50	0.00	1.00	−1.00	621.47	2.00	3.50	4.29	−3.98	613.88	−0.86	4.00	0.89	100.00
**49**	372.50	0.00	1.00	−1.00	621.47	2.00	3.50	4.29	−3.98	613.88	−0.86	4.00	0.89	100.00
**50**	402.53	0.00	1.00	−1.00	647.58	2.00	4.25	4.54	−3.92	1026.72	−0.72	5.00	0.89	100.00
**51**	388.50	0.00	1.00	−2.00	653.04	2.00	3.25	4.34	−4.28	283.29	−1.31	5.00	0.98	96.23
**52**	356.50	0.00	1.00	0.00	612.17	1.00	2.75	5.02	−4.10	1659.62	−0.39	3.00	1.10	100.00
**53**	386.53	0.00	1.00	0.00	650.86	1.00	3.50	5.17	−4.12	1806.41	−0.44	4.00	1.13	100.00
**54**	372.50	0.00	0.00	1.00	600.93	1.00	3.00	5.10	−3.72	5101.41	0.12	5.00	1.05	100.00
**55**	356.50	0.00	0.00	0.00	617.64	1.00	2.25	5.33	−3.98	3852.76	−0.01	5.00	1.21	100.00
**56**	386.53	2.00	1.00	−2.00	824.22	1.00	3.50	6.38	−5.91	1254.62	−1.22	11.00	1.43	100.00
**57**	390.52	0.00	1.00	−2.00	671.57	3.00	4.25	3.88	−4.50	304.62	−1.33	4.00	0.76	94.12
**58**	372.50	0.00	1.00	−2.00	663.72	2.00	3.50	4.42	−4.62	458.92	−1.09	6.00	0.97	100.00
**59**	356.50	1.00	1.00	0.00	652.58	1.00	2.75	5.16	−4.72	1254.20	−0.58	5.00	1.20	100.00
**60**	388.50	0.00	1.00	−2.00	670.71	2.00	3.25	4.39	−4.54	239.87	−1.44	7.00	1.02	95.22
**61**	388.50	1.00	1.00	−1.00	659.93	1.00	3.25	4.69	−4.40	510.35	−0.91	3.00	1.19	100.00
**62**	314.47	1.00	0.00	1.00	559.04	1.00	1.50	4.90	−4.01	3001.20	0.04	2.00	1.11	100.00
**63**	314.47	2.00	0.00	1.00	563.25	1.00	1.50	4.90	−4.04	3001.81	0.03	2.00	1.11	100.00
**64**	372.50	0.00	1.00	−1.00	627.45	2.00	3.50	4.18	−4.07	621.36	−0.77	3.00	0.93	100.00
**65**	355.48	1.00	0.00	0.00	569.69	2.00	3.50	3.91	−3.82	1051.25	−0.53	5.00	0.67	100.00
**66**	623.92	4.00	0.00	0.00	896.54	2.00	5.25	8.42	−5.21	1691.25	−0.66	8.00	2.37	100.00
**67**	623.92	5.00	0.00	−1.00	906.24	2.00	5.25	8.43	−5.35	1545.04	−0.71	9.00	2.38	100.00
**68**	624.90	4.00	0.00	0.00	761.51	1.00	5.25	7.56	−3.60	1130.21	−0.65	7.00	2.07	100.00
**69**	623.92	4.00	0.00	0.00	896.54	2.00	5.25	8.42	−5.21	1691.25	−0.66	8.00	2.37	100.00
**70**	623.92	4.00	0.00	−1.00	905.65	2.00	5.25	8.43	−5.30	1511.67	−0.72	8.00	2.39	100.00
**71**	362.47	0.00	1.00	−1.00	617.02	0.00	6.45	3.05	−3.77	832.11	−0.73	2.00	0.08	100.00
**72**	362.47	0.00	1.00	−1.00	621.09	0.00	6.45	2.84	−4.05	724.55	−0.84	2.00	0.01	94.77
**73**	404.50	0.00	2.00	0.00	650.04	0.00	6.50	3.49	−3.92	925.43	−0.60	3.00	0.35	100.00
**74**	404.50	0.00	2.00	0.00	643.97	0.00	6.50	3.55	−3.76	1033.49	−0.53	3.00	0.36	100.00
**75**	404.50	0.00	2.00	0.00	656.21	0.00	6.50	3.53	−4.01	893.31	−0.63	3.00	0.37	100.00
**76**	404.50	0.00	2.00	0.00	616.22	0.00	6.50	3.46	−3.36	1469.50	−0.35	3.00	0.27	100.00
**77**	400.60	0.00	1.00	0.00	671.71	0.00	4.70	5.26	−3.91	1954.56	−0.51	6.00	0.93	100.00
**78**	400.60	0.00	1.00	0.00	691.19	0.00	4.70	5.44	−4.05	1954.69	−0.53	6.00	1.02	100.00
**79**	432.64	0.00	1.00	0.00	718.22	0.00	5.45	5.47	−4.13	1939.73	−0.66	4.00	0.91	100.00
**80**	432.64	0.00	1.00	0.00	750.60	0.00	5.45	5.72	−4.49	1950.56	−0.70	4.00	1.03	100.00
**81**	390.52	0.00	0.00	0.00	653.42	1.00	5.50	4.27	−4.18	1628.90	−0.49	4.00	0.71	100.00
**82**	390.52	0.00	0.00	0.00	634.28	1.00	5.50	4.08	−3.84	1090.57	−0.62	4.00	0.69	100.00
**83**	390.52	0.00	0.00	0.00	618.00	1.00	5.50	4.05	−3.47	1475.16	−0.47	4.00	0.66	100.00
**84**	390.52	0.00	0.00	0.00	601.76	1.00	5.50	3.92	−3.22	1276.69	−0.50	4.00	0.62	100.00
**85**	404.50	1.00	1.00	0.00	640.06	1.00	7.20	3.48	−3.99	1408.26	−0.59	3.00	0.32	100.00
**86**	390.52	0.00	0.00	0.00	686.36	1.00	5.50	4.45	−4.67	1597.54	−0.56	5.00	0.78	100.00
**87**	250.38	1.00	0.00	−1.00	476.68	1.00	2.00	3.84	−1.14	441.09	−0.18	2.00	0.34	96.78

**Abbreviations:** molecular weight (mol_MW), drug-likeness (#Stars), total solvent accessible surface area (SASA), number of hydrogen bond donors and acceptors (donorHB and acceptHB), predicted octanol-water partitioning (QPlogPo/w), estimated binding to human serum albumin (QPlogKhsa), number of the possible metabolites (# metab), predicted blood-brain partitioning (QPlogBB), percentage of human oral absorption, predicted IC_50_ for inhibiting HERG-K^+^ channels (QPogHERG), predicted apparent Caco-2 cell permeability in nm/s for gut-blood barrier (QPPCaco), central nervous system activity (CNS), number of reactive functional groups present (#rtvFG), and percent human oral absorption.

## Data Availability

The data presented in this study are available in the article.

## Supplementary Materials

The following supporting information can be downloaded at: https://www.mdpi.com/article/10.3390/molecules28031292/s1, Table S1: In-silico docking results of sesquiterpene metabolites with hDHFR (PDB: 1KMV) compared to the reference inhibitor **SRI-9662**. In addition to Simulation Interactions Diagram Report for the following compounds **SRI-9662** (native ref.), **34**, **28**, and **44.**

## References

[1] Osorio E., Aguilera C., Naranjo N., Marín M., Muskus C. (2013). Biochemical characterization of the bifunctional enzyme dihydrofolate reductase-thymidylate synthase from Leishmania (Viannia) and its evaluation as a drug target. Biomedica.

[2] Tobias A.M., Toska D., Lange K., Eck T., Bhat R., Janson C.A., Rotella D.P., Gubler U., Goodey N.M. (2018). Expression, purification, and inhibition profile of dihydrofolate reductase from the filarial nematode Wuchereria bancrofti. PLoS ONE.

[3] Raimondi M.V., Randazzo O., La Franca M., Barone G., Vignoni E., Rossi D., Collina S. (2019). DHFR Inhibitors: Reading the Past for Discovering Novel Anticancer Agents. Molecules.

[4] Hawser S., Lociuro S., Islam K. (2006). Dihydrofolate reductase inhibitors as antibacterial agents. Biochem. Pharmacol..

[5] Rana R.M., Rampogu S., Zeb A., Son M., Park C., Lee G., Yoon S., Baek A., Parameswaran S., Park S.J. (2019). In Silico Study Probes Potential Inhibitors of Human Dihydrofolate Reductase for Cancer Therapeutics. J. Clin. Med..

[6] Srinivasan B., Tonddast-Navaei S., Roy A., Zhou H., Skolnick J. (2019). Chemical space of Escherichia coli dihydrofolate reductase inhibitors: New approaches for discovering novel drugs for old bugs. Med. Res. Rev..

[7] Tibon N.S., Ng C.H., Cheong S.L. (2020). Current progress in antimalarial pharmacotherapy and multi-target drug discovery. Eur. J. Med. Chem..

[8] Giletti A., Esperon P. (2018). Genetic markers in methotrexate treatments. Pharm. J..

[9] Wróbel A., Arciszewska K., Maliszewski D., Drozdowska D. (2020). Trimethoprim and other nonclassical antifolates an excellent template for searching modifications of dihydrofolate reductase enzyme inhibitors. J. Antibiot..

[10] Chawla P., Teli G., Gill R.K., Narang R.K. (2021). An Insight into Synthetic Strategies and Recent Developments of Dihydrofolate Reductase Inhibitors. ChemistrySelect.

[11] Kreutzfeld O., Tumwebaze P.K., Byaruhanga O., Katairo T., Okitwi M., Orena S., Rasmussen S.A., Legac J., Conrad M.D., Nsobya S.L. (2022). Decreased Susceptibility to Dihydrofolate Reductase Inhibitors Associated With Genetic Polymorphisms in Ugandan Plasmodium falciparum Isolates. J. Infect. Dis..

[12] Krucinska J., Lombardo M.N., Erlandsen H., Estrada A., Si D., Viswanathan K., Wright D.L. (2022). Structure-guided functional studies of plasmid-encoded dihydrofolate reductases reveal a common mechanism of trimethoprim resistance in Gram-negative pathogens. Commun. Biol..

[13] Arya H., Coumar M.S., Bhatt T.K., Nimesh S. (2021). Chapter 4—Lead identification and optimization. The Design & Development of Novel Drugs and Vaccines.

[14] Aguayo-Ortiz R., Fernández-de Gortari E., Medina-Franco J.L. (2016). Chapter 2—Overview of Computer-Aided Drug Design for Epigenetic Targets. Epi-Informatics.

[15] Nastrucci C., Cesario A., Russo P. (2012). Anticancer Drug Discovery from the Marine Environment. Recent Pat. Anti-Cancer Drug Discov..

[16] Abdelmohsen U.R., Balasubramanian S., Oelschlaeger T.A., Grkovic T., Pham N.B., Quinn R.J., Hentschel U. (2017). Potential of marine natural products against drug-resistant fungal, viral, and parasitic infections. Lancet Infect. Dis..

[17] Lu W.-Y., Li H.-J., Li Q.-Y., Wu Y.-C. (2021). Application of marine natural products in drug research. Bioorganic Med. Chem..

[18] Pereira F., Aires-de-Sousa J. (2018). Computational Methodologies in the Exploration of Marine Natural Product Leads. Mar. Drugs.

[19] Chen G., Seukep A.J., Guo M. (2020). Recent Advances in Molecular Docking for the Research and Discovery of Potential Marine Drugs. Mar. Drugs.

[20] Marcos I.S., Conde A., Moro R.F., Basabe P., Diez D., Urones J.G. (2010). Quinone/Hydroquinone Sesquiterpenes. Mini-Rev. Org. Chem..

[21] Kumar M., Dagar A., Gupta V.K., Sharma A. (2014). In silico docking studies of bioactive natural plant products as putative DHFR antagonists. Med. Chem. Res..

[22] Herrmann F.C., Sivakumar N., Jose J., Costi M.P., Pozzi C., Schmidt T.J. (2017). In Silico Identification and In Vitro Evaluation of Natural Inhibitors of Leishmania major Pteridine Reductase I. Molecules.

[23] Jose S., Devi S.S., Al-Khafaji K. (2022). Phytochemical constituents of Inula britannica as potential inhibitors of dihydrofolate reductase: A strategic approach against shigellosis. J. Biomol. Struct. Dyn..

[24] Possart K., Herrmann F.C., Jose J., Costi M.P., Schmidt T.J. (2022). Sesquiterpene Lactones with Dual Inhibitory Activity against the Trypanosoma brucei Pteridine Reductase 1 and Dihydrofolate Reductase. Molecules.

[25] Herrera-Acevedo C., Monroy-Velandia D., Flores-Gaspar A., Coy-Barrera E. In Silico Studies to Evaluate Interactions Between Kaurane-Type Diterpenes and the Dihy-drofolate Reductase–Thymilidine Synthase of Three Leishmania Species. Proceedings of the International Conference on Multidisciplinary Sciences (MOL2NET 2018), 4th edition UFPB.

[26] Kwon Y.-J., Sohn M.-J., Kim H.-J., Kim W.-G. (2014). The Lactone form of stachybotrydial: A new inhibitor of dihydrofolate reductase from stachybotrys sp. FN298. Biol. Pharm. Bull..

[27] Alea G., Carroll A.R., Bowden B.F. (1994). Coscinoquinol, a New Cytotoxic Sesterterpene From a Dictyoceratid Sponge, Coscinoderma sp.. Aust. J. Chem..

[28] Zubía E., Ortega M.J., Luis Carballo J., Salvá J. (1994). Sesquiterpene hydroquinones from the sponge Reniera mucosa. Tetrahedron.

[29] Ibrahim S.R.M., Fadil S.A., Fadil H.A., Hareeri R.H., Alolayan S.O., Abdallah H.M., Mohamed G.A. (2022). Dactylospongia elegans—A Promising Drug Source: Metabolites, Bioactivities, Biosynthesis, Synthesis, and Structural-Activity Relationship. Mar. Drugs.

[30] Klon A.E., Héroux A., Ross L.J., Pathak V., Johnson C.A., Piper J.R., Borhani D.W. (2002). Atomic Structures of Human Dihydrofolate Reductase Complexed with NADPH and Two Lipophilic Antifolates at 1.09Å and 1.05Å Resolution. J. Mol. Biol..

[31] Olsson M.H.M., Søndergaard C.R., Rostkowski M., Jensen J.H. (2011). PROPKA3: Consistent Treatment of Internal and Surface Residues in Empirical pKa Predictions. J. Chem. Theory Comput..

[32] (2021). LigPrep.

[33] Friesner R.A., Banks J.L., Murphy R.B., Halgren T.A., Klicic J.J., Mainz D.T., Repasky M.P., Knoll E.H., Shelley M., Perry J.K. (2004). Glide:  A New Approach for Rapid, Accurate Docking and Scoring. 1. Method and Assessment of Docking Accuracy. J. Med. Chem..

[34] Friesner R.A., Murphy R.B., Repasky M.P., Frye L.L., Greenwood J.R., Halgren T.A., Sanschagrin P.C., Mainz D.T. (2006). Extra Precision Glide:  Docking and Scoring Incorporating a Model of Hydrophobic Enclosure for Protein−Ligand Complexes. J. Med. Chem..

[35] (2021). Glide.

[36] Hou T., Wang J., Li Y., Wang W. (2011). Assessing the Performance of the MM/PBSA and MM/GBSA Methods. 1. The Accuracy of Binding Free Energy Calculations Based on Molecular Dynamics Simulations. J. Chem. Inf. Model..

[37] Koushki E.H., Abolghasemi S., Mollica A., Aghaeepoor M., Moosavi S.S., Farshadfar C., Hasanpour B., Feyzi B., Abdi F., Mirzaie S. (2020). Structure-based virtual screening, molecular docking and dynamics studies of natural product and classical inhibitors against human dihydrofolate reductase. Netw. Model. Anal. Health Inform. Bioinform..

[38] (2021). QikProp.

[39] (2021). Maestro.

[40] (2021). Desmond Molecular Dynamics System, Maestro-Desmond Interoperability Tools.

[41] Hollingsworth S.A., Dror R.O. (2018). Molecular Dynamics Simulation for All. Neuron.

[42] Leelananda S.P., Lindert S. (2016). Computational methods in drug discovery. Beilstein J. Org. Chem..

